# Author Correction: Targeting ferroptosis protects against experimental (multi)organ dysfunction and death

**DOI:** 10.1038/s41467-025-64122-6

**Published:** 2025-09-18

**Authors:** Samya Van Coillie, Emily Van San, Ines Goetschalckx, Bartosz Wiernicki, Banibrata Mukhopadhyay, Wulf Tonnus, Sze Men Choi, Ria Roelandt, Catalina Dumitrascu, Ludwig Lamberts, Geert Dams, Wannes Weyts, Jelle Huysentruyt, Behrouz Hassannia, Irina Ingold, Suhas Lele, Evelyne Meyer, Maya Berg, Ruth Seurinck, Yvan Saeys, An Vermeulen, Alexander L. N. van Nuijs, Marcus Conrad, Andreas Linkermann, Mohan Rajapurkar, Peter Vandenabeele, Eric Hoste, Koen Augustyns, Tom Vanden Berghe

**Affiliations:** 1 VIB-UGent Center for Inflammation Research, Ghent, Belgium; 2 Department of Biomedical Molecular Biology, Ghent University, Ghent, Belgium; 3 Department of Biomedical Sciences, University of Antwerp, Antwerp, Belgium; 4 Department of Nephrology, Muljibhai Patel Society for Research in Nephro-Urology, Nadiad, India; 5 Department of Internal Medicine 3, University Hospital Carl Gustav Carus, the Technische Universität Dresden, Dresden, Germany; 6 Department of Applied Mathematics, Computer Science and Statistics, Ghent University, Ghent, Belgium; 7 Department of Pharmaceutical Sciences, Toxicological Centre, University of Antwerp, Antwerp, Belgium; 8 VIB-UGent Center for Medical Biotechnology, Ghent, Belgium; 9 Institute of Metabolism and Cell Death, Helmholtz Zentrum München, German Research Center for Environmental Health, Munich, Germany; 10 Department of Medicine III, Klinikum rechts der Isar, Technical University of Munich, Munich, Germany; 11 Department of Pharmacology, Toxicology and Biochemistry, Ghent University, Merelbeke, Belgium; 12 Department of Bioanalysis, Ghent University, Ghent, Belgium; 13 National Research Medical University, Laboratory of Experimental Oncology, Moscow, Russia; 14 Biotechnology Center, Technische Universität Dresden, Dresden, Germany; 15 Methusalem program, Ghent University, Ghent, Belgium; 16 Intensive Care Unit, Ghent University Hospital; Ghent University, Ghent, Belgium

**Keywords:** Cell death, Translational research, Preclinical research, Diseases

Correction to: *Nature Communications* 10.1038/s41467-022-28718-6, published online 24 February 2022

In the version of the article initially published, due to a calibration error, values for malondialdehyde (MDA) concentrations reported in the Results, Methods, Fig. 1 and Supp Figs. 1, 2 were underestimated by a factor of five. In the first paragraph of the Results, “…MDA^max^ > 14.25 μM” and “…MDA^max^ of 16.9 μM” replace the original “MDA^max^ > 2.85 μM” and “MDA^max^ of 3.38 μM,” respectively, while in the first paragraph of the “Statistical analysis” section of Methods, “…MDA^max^ < 14.25 μM resp. 16.9 versus ≥ 14.25 resp. 16.9 μM” now replaces the original “MDA^max^ < 2.85 μM resp. 3.38 versus ≥ 2.85 resp. 3.38 μM.” In Fig. 1h, i, “14.25 μМ” replaces “2.85 μM” in three places. These corrections do not alter the results, interpretation or conclusions of the study. The text, Fig. 1 and Supplementary Figs. 1, 2 are now updated in the HTML and PDF versions of the article.

