# Supplementary material for: Targeting ferroptosis protects against experimental (multi)organ dysfunction and death
Source: Nat Commun. 2022 Feb 24;13:1046. doi: 10.1038/s41467-022-28718-6 (PMC8873468; doi:10.1038/s41467-022-28718-6)
Supplement: Supplementary file 1 — Supplementary Information [file 41467_2022_28718_MOESM1_ESM.pdf]

Supplementary figures

Targeting ferroptosis protects against experimental (multi)organ dysfunction and death.

Samya Van Coillie[1,2], Emily Van San[1,2], Ines Goetschalckx[3], Bartosz Wiernicki[1,2], Banibrata Mukhopadhyay[4], Wulf Tonnus[5], Sze Men Choi[1,2], Ria Roelandt[1,2,6], Catalina Dumitrascu[7], Ludwig Lamberts[3], Geert Dams[3], Wannes Weyts[8], Jelle Huysentruyt[1,2], Behrouz Hassannia[1,2], Irina Ingold[9,10], Suhas Lele[4], Evelyne Meyer[11], Maya Berg[7], Ruth Seurinck[1,6], Yvan Saeys[1,6], An Vermeulen[12], Alexander L.N. van Nuijs[7], Marcus Conrad[9,13], Andreas Linkermann[5,14], Mohan Rajapurkar[4], Peter Vandenabeele[1,2,15], Eric Hoste[16], Koen Augustyns[7] and Tom Vanden Berghe[1,2,3].

[1]VIB-UGent Center for Inflammation Research; Ghent, Belgium.

[2]Department of Biomedical Molecular Biology, Ghent University; Ghent, Belgium.

[3]Department of Biomedical Sciences; University of Antwerp, Antwerp, Belgium.

[4]Department of Nephrology, Muljibhai Patel Society for Research in Nephro-Urology; Nadiad, India.

[5]Department of Internal Medicine 3, University Hospital Carl Gustav Carus, the Technische Universität Dresden; Dresden, Germany.

[6]Department of Applied Mathematics, Computer Science and Statistics, Ghent University; Ghent, Belgium.

[7]Department of Pharmaceutical Sciences, Toxicological Centre, University of Antwerp; Antwerp, Belgium.

[8]VIB-UGent Center for Medical Biotechnology; Ghent, Belgium.

[9]Institute of Metabolism and Cell Death, Helmholtz Zentrum München, German Research Center for Environmental Health; Munich, Germany.

[10]Department of Medicine III, Klinikum rechts der Isar, Technical University of Munich; Munich, Germany.

[11]Department of Pharmacology, Toxicology and Biochemistry, Ghent University; Merelbeke, Belgium.

[12]Department of Bioanalysis, Ghent University; Ghent, Belgium.

[13]National Research Medical University, Laboratory of Experimental Oncology; Moscow, Russia.

[14]Biotechnology Center, Technische Universität Dresden; Dresden, Germany.

[15]Methusalem program, Ghent University; Ghent, Belgium.

[16]Intensive Care Unit, Ghent University Hospital; Ghent, Belgium.

Correspondence: Prof. Tom Vanden Berghe – tom.vandenberghe@uantwerp.be

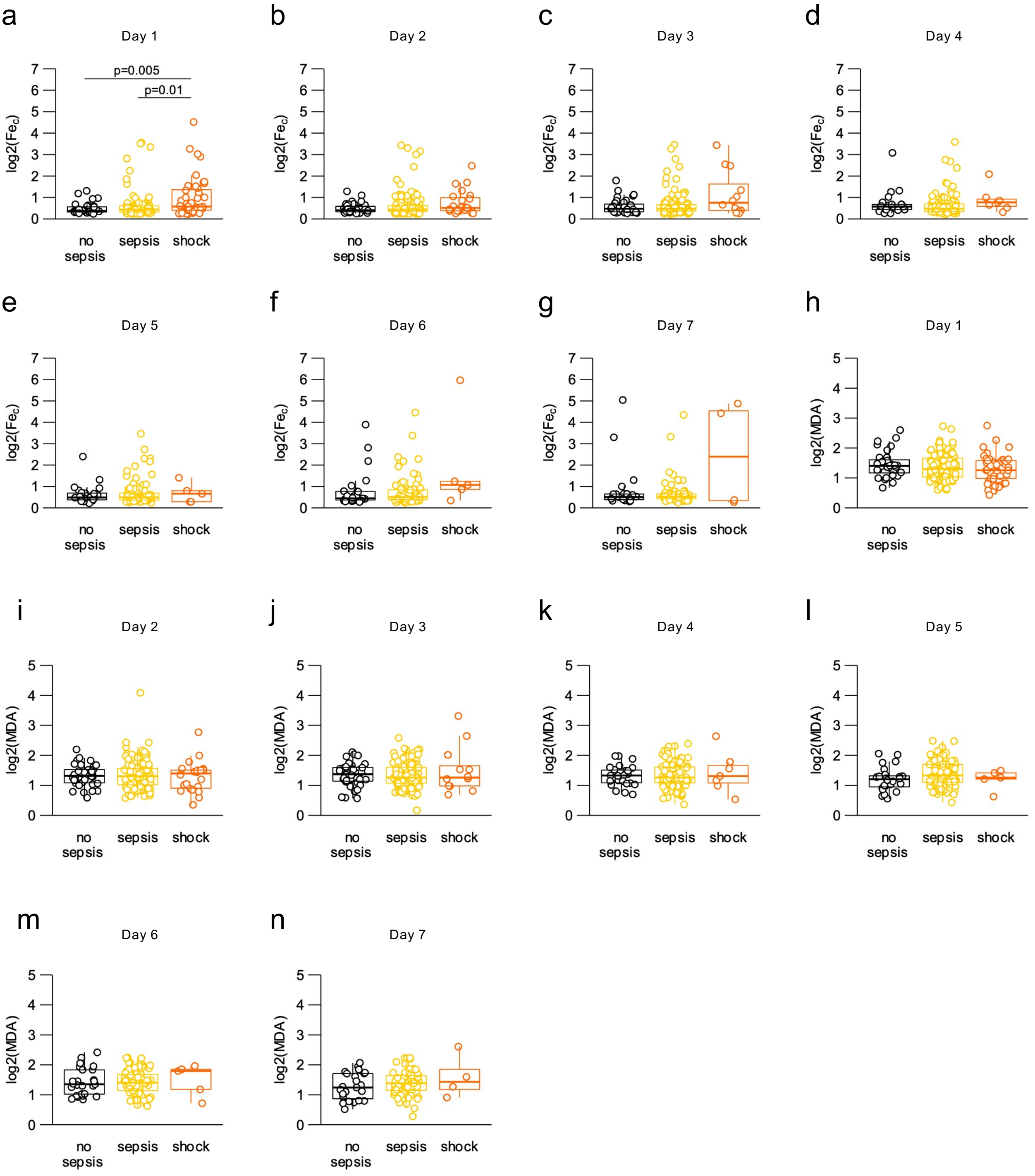

Supplementary Fig. 1: Patients with sepsis cannot be distinguished from non-septic patients based on plasma catalytic iron (Fe_c) or MDA levels.

a-g Boxplots showing the log-transformed catalytic iron (Fe_c) values from day 1 to 7 of all patients grouped by the presence of sepsis, septic shock or non-septic MODS (n for day 1 to 7 = 178,169,158,143,127,110 and 96). **h-n** Boxplots showing the log-transformed malondialdehyde (MDA) values from day 1 to 7 of all patients grouped by the presence of sepsis, septic shock or non-septic MODS (n for day 1 to 7 = 178,168,158,143,127,110 and 96). Data were analyzed using the Kruskal-Wallis (omnibus) and two-sided Wilcoxon-Mann-Whitney (pairwise) test (**a**-**n**). The full range of observations are plotted on all the boxplots, where the bottom, center and top of the bounding box represent respectively the 25th, 50th and 75th percentiles. The whiskers extend to a maximum of 1.5 times the interquartile range (IQR = Q3 − Q1) beyond the bounding box.

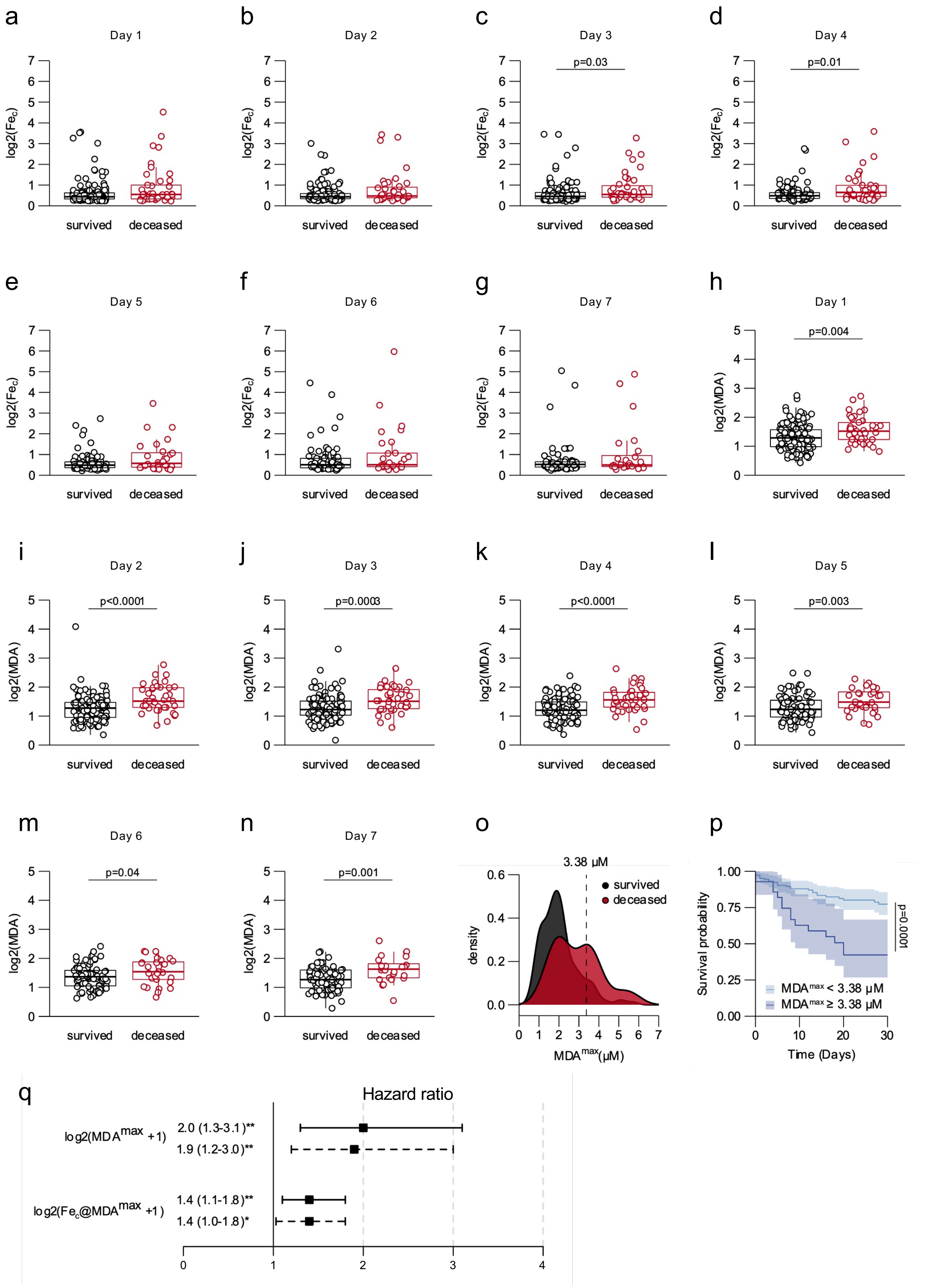

Supplementary Fig. 2: MDA has higher prognostic value for 30-day survival than catalytic iron.
Legend on next page

Supplementary Fig. 2: MDA has higher prognostic value for 30-day survival than catalytic iron.
a-**g** Boxplots showing the log-transformed catalytic iron (Fe_c) values from day 1 to 7 of the patients who deceased and those who survived (n for day 1 to 7 = 178,169,158,143,127,110 and 96). **h-n**, Boxplots showing the log-transformed malondialdehyde (MDA) values from day 1 to 7 of the patients who deceased and those who survived (n for day 1 to 7 = 178,168,158,143,127,110 and 96). **o**, Histogram representing the density distribution of MDA^{max} values for the patients who survived a 30-day survival follow-up (black) and those who did not (red). **p**, Survival curves representing the patients with values of $MDA^{max} < 3.38$ µM (light blue) and $MDA^{max} \geq 3.38$ µM (dark blue) with n = 176. **q**, Forest plot summary representing the hazard ratio, $CI_{95\%}$ and associated p-value for the unadjusted predictors MDA^{max} level and corresponding Fe_c (full line) and for the predictors MDA^{max} level and corresponding Fe_c adjusted for age and SOFA score (dashed line). Data were analyzed using a two-sided Wilcoxon–Mann–Whitney test (**a**-**n**), two-sided log-Rank test (**p**) and cox proportional hazards model (**q**). The full range of observations are plotted on all the boxplots, where the bottom, center and top of the bounding box represent respectively the 25th, 50th and 75th percentiles. The whiskers extend to a maximum of 1.5 times the interquartile range (IQR = Q3 − Q1) beyond the bounding box. Source data are provided as a Source Data file.

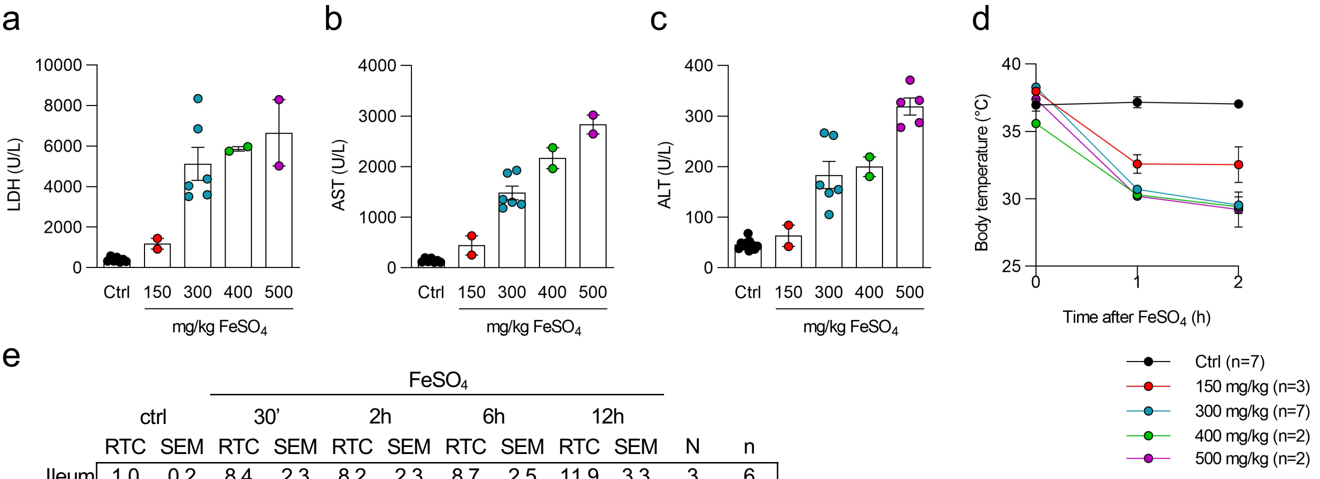

e

Relative iron level

	ctrl		FeSO4 30'		FeSO4 2h		FeSO4 6h		FeSO4 12h			
	RTC	SEM	RTC	SEM	RTC	SEM	RTC	SEM	RTC	SEM	N	n
Ileum	1.0	0.2	8.4	2.3	8.2	2.3	8.7	2.5	11.9	3.3	3	6
Liver	1.0	0.0	2.1	0.2	2.3	0.2	2.9	0.2	3.4	0.2	3	6
Kidney	1.0	0.0	1.9	0.1	1.8	0.1	1.6	0.1	2.0	0.3	3	6
Spleen	1.0	0.0	1.3	0.2	1.2	0.1	1.2	0.2	2.1	0.2	3	6
Brain	1.0	0.0	1.1	0.1	1.2	0.2	1.4	0.2	1.4	0.1	3	6
Lung	1.0	0.1	1.2	0.1	1.1	0.1	1.1	0.1	1.5	0.3	3	6
Muscle	1.0	0.1	0.7	0.2	1.0	0.2	0.8	0.2	0.9	0.3	3	6
Heart	1.0	0.1	0.9	0.1	1.0	0.1	0.9	0.1	1.1	0.1	3	6
Plasma	1.0	0.1	7.7	1.3	4.3	0.2	2.1	0.2	2.0	0.1	3	5-6

f

Relative level in plasma

	ctrl		FeSO4 30'		FeSO4 1h		FeSO4 2h		FeSO4 4h		FeSO4 6h		FeSO4 12h			
	RTC	SEM	RTC	SEM	RTC	SEM	RTC	SEM	RTC	SEM	RTC	SEM	RTC	SEM	N	n
CK	1.0	0.1	7.5	1.8	9.8	1.2	16.3	3.3	30.5	4.6	66.4	11.9	84.3	7.6	5	6-10
LDH	1.0	0.0	2.6	0.5	3.9	0.4	5.9	1.3	12.5	1.8	30.6	5.6	58.7	10.0	5	6-10
AST	1.0	0.1	3.8	0.7	5.2	0.4	6.8	0.8	9.7	1.1	10.8	1.1	13.1	1.3	5	6-10
ALT	1.0	0.0	1.7	0.2	2.1	0.1	2.8	0.3	4.7	0.8	4.0	0.3	7.0	1.4	5	6-9
Trop T	1.0	0.1	2.5	1.1	2.3	0.3	1.8	0.2	2.6	0.2	3.3	0.2	4.9	1.2	6	6-8
Cr	1.0	0.0	1.2	0.1	1.0	0.0	2.5	0.2	2.4	0.3	2.5	0.5	1.8	0.4	6	8-13
Urea	1.0	0.0	1.3	0.0	1.4	0.1	1.8	0.0	2.0	0.2	2.4	0.2	2.2	0.3	4	6-9
Mb	1.0	0.1	1.4	0.1	1.2	0.1	1.8	0.4	1.3	0.2	1.2	0.1	1.0	0.2	4	6-10
Ferritin	1.0	0.0	1.0	0.0	1.3	0.1	1.1	0.0	1.4	0.1	1.4	0.1	1.5	0.1	3	6-7

g

Relative MDA level

	ctrl		FeSO4 30'		FeSO4 1h		FeSO4 2h		FeSO4 4h		FeSO4 6h		FeSO4 12h			
	RTC	SEM	RTC	SEM	RTC	SEM	RTC	SEM	RTC	SEM	RTC	SEM	RTC	SEM	N	n
Kidney	1.0	0.1	4.2	0.5	4.4	0.8	4.0	0.7	4.4	0.6	3.0	0.4	3.1	0.5	3	5-6
Liver	1.0	0.0	3.6	1.1	3.4	1.0	3.3	0.6	3.4	1.3	2.9	0.9	3.2	1.5	3	5-6
Ileum	1.0	0.1	2.3	0.3	1.4	0.2	1.7	0.3	1.5	0.2	1.4	0.1	1.5	0.2	3	5-8
Muscle	1.0	0.1	1.4	0.2	1.1	0.2	1.1	0.2	1.4	0.2	1.1	0.1	1.2	0.3	3	6-8
Spleen	1.0	0.0	1.2	0.2	1.0	0.3	0.8	0.1	1.5	0.4	1.3	0.2	1.2	0.3	3	5-6
Heart	1.0	0.2	1.0	0.1	0.7	0.1	0.8	0.0	1.0	0.2	1.4	0.2	1.3	0.1	3	5-6
Brain	1.0	0.1	1.0	0.0	1.2	0.2	1.0	0.1	1.0	0.1	1.0	0.1	0.9	0.1	3	5-8
Lung	1.0	0.1	0.9	0.1	0.6	0.1	0.6	0.1	0.7	0.1	0.6	0.1	0.6	0.1	3	5-6
Plasma	1.0	0.1	1.8	0.2	1.7	0.2	1.6	0.2	1.4	0.1	1.6	0.3	1.0	0.1	3	5-8

Supplementary Fig. 3: Dose optimization and characterization of iron sulphate as iron overload model.

a-c Plasma levels of LDH, AST and ALT 2h after injection of different dosages of iron sulphate ranging from 150 to 500 mg/kg (n/condition from left to right is as follows: n = 7, 2, 6, 2, 2 for LDH, AST; n = 11, 2, 6, 2, 5 for ALT). **d** Body temperature 2h after injection of different dosages of iron sulphate ranging from 150 to 500 mg/kg. **e-g** Tables representing the relative iron levels, plasma damage markers and MDA levels after acute iron overload as a function of time. N represents the number of independently performed experiments; n represents the total number of mice per time point. RTC, relative to control; SEM, standard error of the mean. Source data are provided as a Source Data file.

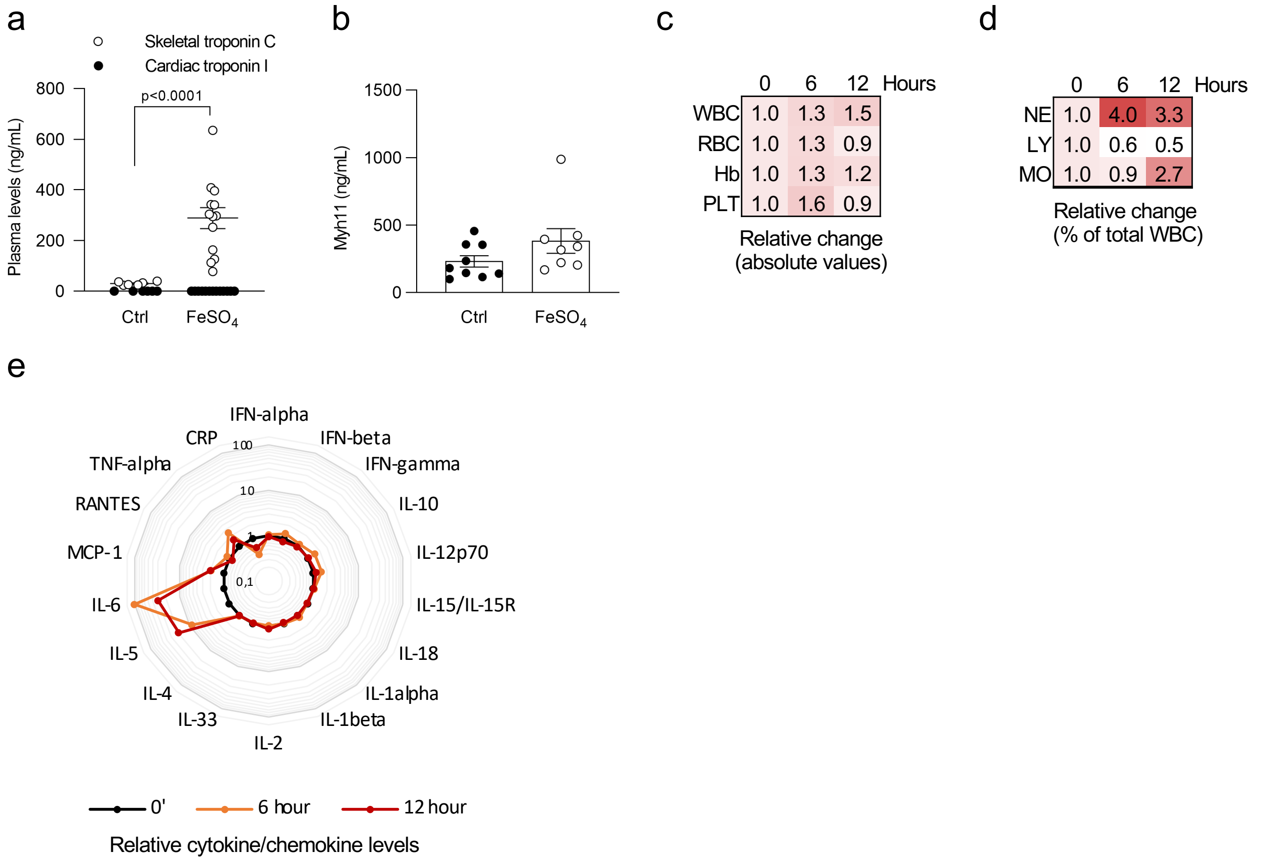

Supplementary Fig. 4: Acute iron overload causes skeletal muscle injury, neutrophilia, lymphopenia and elevated levels of plasma IL-5 and -6.

a Plasma levels of skeletal troponin C (total n = 6 for ctrl group; total n = 13 for $FeSO_4$ group) and cardiac troponin I (total n = 6 for ctrl group; total n = 14 for $FeSO_4$ group) 2h after iron overload. The combined results of 2 independent experiments are shown. **b** Plasma levels of Myosin heavy chain 11 (Myh11) 2h after iron overload, reflecting smooth muscle injury. The combined results of minimum 2 independent experiments are shown (total n = 9 for ctrl group; total n = 8 for $FeSO_4$ group). **c**, **d** Heatmaps representing the relative blood count and the relative change in WBC populations after iron overload as a function of time. The combined results of 3 independent experiments are shown (total n = 7 for 0h, 6h; total n = 6 for 12h). **e** Radar web representing the relative cytokine and chemokine levels after iron overload as a function of time. The combined results of 3 independent experiments are shown (total n = 6 for 0h; total n = 5 for 6h, 12h). Data were analyzed using unpaired, two-tailed T testing (**a**). Means and SEMs are represented. WBC white blood cell. Source data are provided as a Source Data file.

a

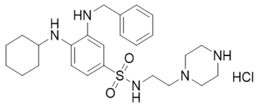

UAMC-3203

b

Solubility at 2mM in 0.9% NaCl

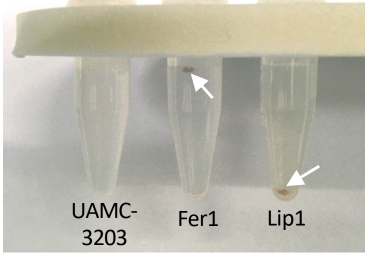

c

Plasma	Rat PK (iv) 4.94 mg kg^{-1}	Mouse PK (iv) 12.35mg kg^{-1}
$T_{1/2}$ (h)	5.10	3.24
V (ml)	7976	762
C_0 (ug ml^{-1})	0.32	1.01
Total Cl (ml h^{-1})	1084	163
Total Cl (ml min^{-1} kg^{-1})	81.0	127
MRT (h)	nd	2.73
Vss (ml)	nd	444
B/P	nd	0.89

d

In vivo PK analysis of UAMC-3203 in mice

	C_0 (µg/mL or µg/g)	k_e (1/hr)	$t_{1/2}$ (hr)	$AUC_{0\text{-}inf}$ (µg*hr/mL or µg*hr/g)	T/P
Plasma	1.01	0.214	3.24	1.34	-
Intestine[b]	5.58	0.176	3.94	40.2	30.0
Kidney	133	0.242	2.86	294	219
Lung	6.00	0.232	2.99	14.0	10.5
Muscle[a,b]	13.0	0.359	1.93	39.8	29.7

[a] $t_{1/2}$ calculated using mean values

[b] The time point at 24h was excluded for the calculation of $t_{1/2}$

In vivo PK analysis of UAMC-3203 in rats

	C_0 (µg/mL or µg/g)	k_e (1/hr)	$t_{1/2}$ (hr)	$AUC_{0\text{-}inf}$ (µg*hr/mL or µg*hr/g)	T/P[a]
Plasma	0.320	0.136	5.10	0.885	-
Intestine	nd	0.149	4.65	nd	nd
Lung	15.09	0.173	4.01	101	114
Muscle	1.45	0.102	6.79	11.4	12.9
Liver	7.07	nd	nd	24.9	28.2
Spleen	2.95	nd	nd	11.1	12.5
Heart	11.8	nd	nd	26.3	29.7

[a] T/P calculated using AUC values up to 12hr

nd: not determined

Supplementary Fig. 5: High solubility and favorable PK properties of Fer-analogue UAMC-3203 in mice and rats.

a Chemical structure of UAMC-3203. **b** Image illustrating solubility at 2mM of UAMC-3203 in 0.9% NaCl, and insolubility of Fer1 and Lip1 (precipitate indicated with arrow). **c** Plasma PK properties of UAMC-3203 in mice or rats after intravenous bolus dosing at 12.35 mg/kg and 4.94mg/kg respectively, showing the elimination half-life ($t_{1/2}$), the volume of distribution (V), the concentration of UAMC-3203 at time 0 (C_0), total systemic clearance (Cl), mean residence time (MRT), steady-state volume of distribution (Vss) and the blood to plasma ratio (B/P) (n = 3/time point) . **d** Table showing the concentration of UAMC-3203 in mice or rats at time 0 (C_0), its elimination rate constant (k_e), its elimination half-life ($t_{1/2}$), its total area under the curve ($AUC_{0\text{-}inf}$) and its tissue to plasma ratio (T/P) in plasma, and different tissues (n = 3/time point) after intravenous bolus dosing at 12.35 mg/kg and 4.94 mg/kg respectively. Source data are provided as a Source Data file.

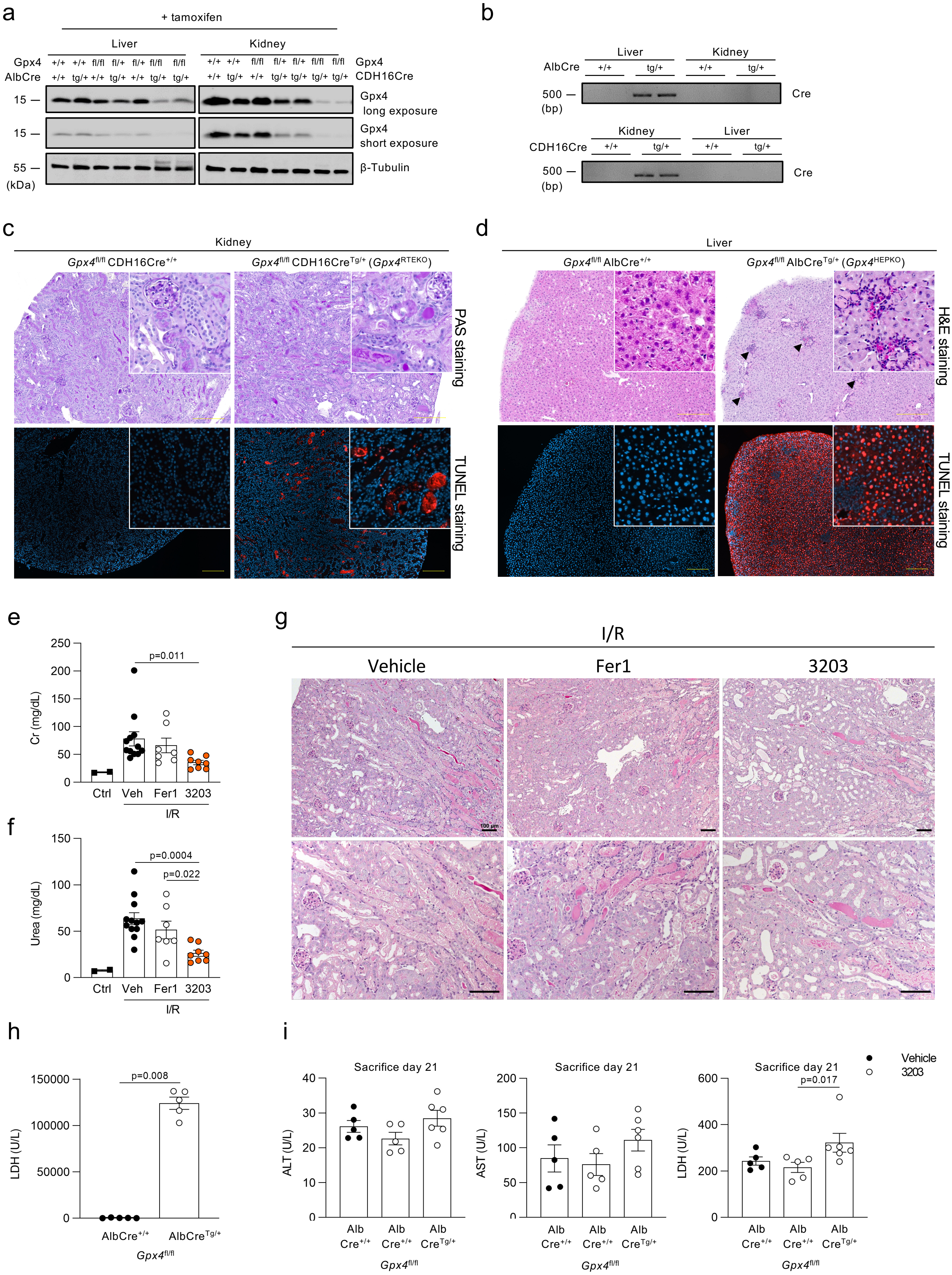

Supplementary Fig. 6: Ferroptosis driven acute liver and kidney injury.
Legend on next page

Supplementary Fig. 6: Ferroptosis driven acute liver and kidney injury.

a Western blot illustrating the knock down of GPX4 in liver tissue of *Gpx4*$^{fl/fl}$AlbCreERT2$^{Tg/+}$ and in kidney tissue of *Gpx4*$^{fl/fl}$CDH16CreERT2$^{Tg/+}$ mice upon TAM administration. The blots shown are representative for 2 independent (*Gpx4*$^{fl/fl}$AlbCreERT2$^{Tg/+}$) and 1 (*Gpx4*$^{fl/fl}$CDH16CreERT2$^{Tg/+}$) experiment. **b** PCR gel with primers binding only upon excision of *Gpx4* performed on liver (vs kidney) tissue of *Gpx4*$^{fl/fl}$AlbCreERT2$^{Tg/+}$ mice and kidney (vs liver) tissue of *Gpx4*$^{fl/fl}$CDH16CreERT2$^{Tg/+}$ mice upon TAM administration. The gels shown are representative for 2 independent (*Gpx4*$^{fl/fl}$AlbCreERT2$^{Tg/+}$) and 1 (*Gpx4*$^{fl/fl}$CDH16CreERT2$^{Tg/+}$) experiment. **c** (Immuno)histochemical staining of kidney tissue of *Gpx4*$^{fl/f}$ CDH16CreERT2$^{Tg/+}$ mice which were sacrificed once a human endpoint was reached, with Periodic acid–Schiff's (PAS; total n/genotype = 5) and TUNEL (total n = 6 for CreERT2$^{+/+}$; total n = 7 for CreERT2$^{Tg/+}$ mice) staining. Photomicrographs representative for the outcome of minimum 2 independent experiments (total n=7/ condition) are shown. Scale bar represents 200 µm. **d** (Immuno)histochemical staining of liver tissue of Gpx4$^{fl/fl}$AlbCreERT2$^{Tg/+}$ mice which were sacrificed once a human endpoint was reached, with H&E (total n/genotype = 6) and TUNEL (total n = 8 for CreERT2$^{+/+}$; total n = 7 for CreERT2$^{Tg/+}$ mice) staining. Arrows indicate inflammatory infiltrates. Photomicrographs representative for the outcome of minimum 2 independent experiments are shown. Scale bar represents 200 µm. **e**, **f** Plasma levels of creatinine (Cr) and urea 48h after ischemia reperfusion for mice treated with vehicle (2% DMSO), Fer1 or compound UAMC-3203 (total n/condition from left to right is as follows: n = 2, 12, 7, 8). This experiment was performed with C57Bl/6N mice provided by Charles River. **g** Histological PAS staining of kidney tissue 48h after ischemia reperfusion for mice treated with vehicle (2% DMSO), Fer1 or compound UAMC-3203 (total n/condition from left to right is as follows: n = 12, 7, 8). Scale bar represents 100 µm. This experiment was performed with C57Bl/6N mice provided by Charles River. **h** Plasma LDH levels of *Gpx4*$^{fl/fl}$AlbCreERT2$^{Tg/+}$ mice which were sacrificed once a human endpoint was reached. The combined results of 2 independent experiments are shown (total n = 5/condition). **i** Plasma levels of ALT, AST and LDH of *Gpx4*$^{fl/fl}$AlbCreERT2$^{Tg/+}$ mice sacrificed on day 21 (16 days after final TAM administration) which were treated daily with vehicle (2% DMSO) or compound UAMC-3203. The combined results of 4 independent experiments are shown (total n/condition from left to right is as follows: n = 5, 5, 6). Data were analyzed using one-way ANOVA followed by pairwise T testing (**e**, **f**) and unpaired, two-tailed T testing (**h**,**i**). Means and SEMs are represented. Source data are provided as a Source Data file.

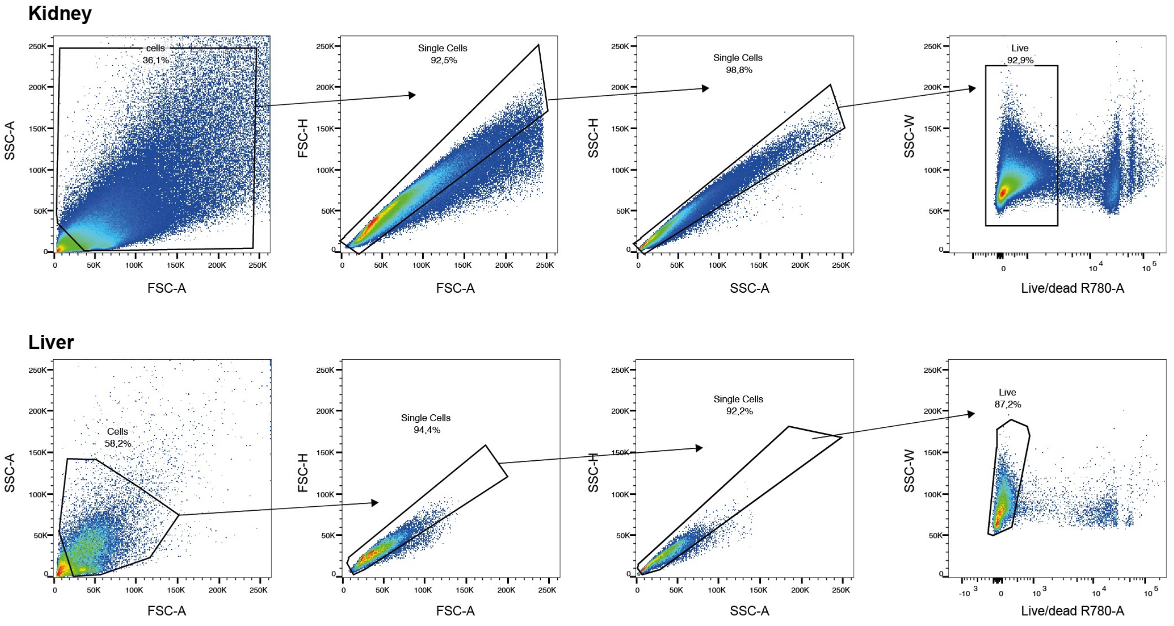

Supplementary Fig. 7: Gating strategy applied to kidney and liver cell suspensions during flow cytometry.

Exclusion of the debris based on the size of the particles was ensured via the FSC-A vs SSC-A plot. FSC-A vs FSC-H and SSC-A and SSC-H plotting was used to exclude cell doublets based on the proportional increase of the area and height of the peak in both forward and side scatter. Finally, dead cells were excluded using the permeability marker (DRAQ7).

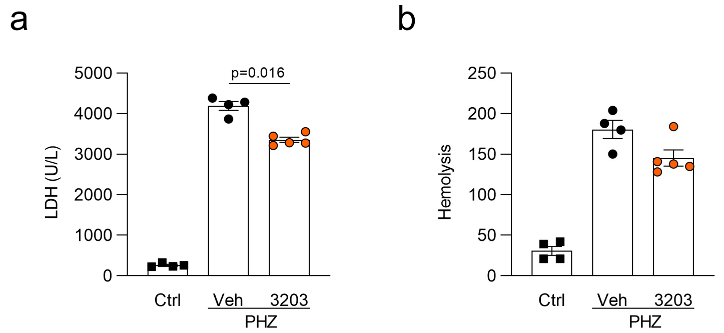

Supplementary Fig. 8: UAMC-3203 provides mild protection against phenylhydrazine induced MODS.

a,**b** Plasma levels of LDH and hemolysis 24h after phenylhydrazine administration for mice treated with vehicle (0,9% NaCl) or compound UAMC-3203. The combined results of 2 independent experiments are shown (total n/condition from left to right is as follows: n = 4, 4, 5). These experiments were performed with mice on a C57BL/6N background bred in-house. Data were analyzed using unpaired two-tailed T testing. Means and SEMs are represented. Source data are provided as a Source Data file.

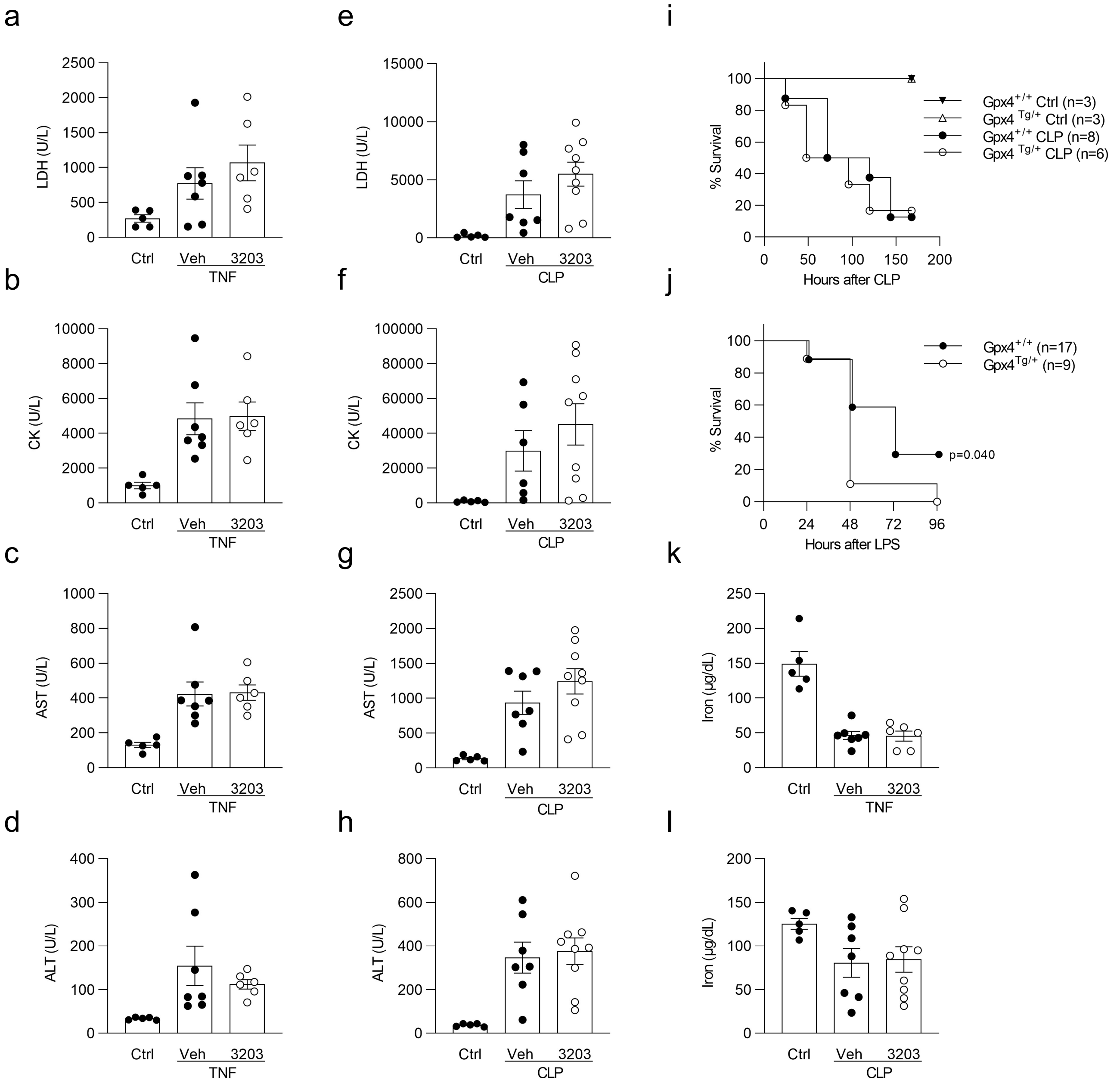

Supplementary Fig. 9: Ferroptosis targeting does not protect against septic shock in mice.
a-**d** Plasma levels of LDH, CK, AST and ALT 8h after TNF challenge for mice treated with vehicle (2% DMSO) or compound UAMC-3203. The combined results of 2 independent experiments are shown (total n/condition from left to right is as follows: n = 5, 7, 6). **e**-**h** Plasma levels of LDH, CK, AST and ALT 24h after CLP for mice treated with vehicle (2% DMSO) or compound UAMC-3203. The combined results of 2 independent experiments are shown (total n/condition from left to right is as follows: n = 5, 7, 9 for LDH, AST, ALT; n = 5, 6, 9 for CK). **i** Survival curve of $Gpx4^{Tg/+}$ mice after CLP. The combined results of 2 independent experiments are shown. **j** Survival curve of $Gpx4^{Tg/+}$ mice after LPS challenge. The combined results of minimum 3 independent experiments are shown. **k** Plasma iron levels 8h after TNF challenge for mice treated with vehicle (2% DMSO) or compound UAMC-3203. The combined results of 2 independent experiments are shown (total n/condition from left to right is as follows: n = 5, 7, 6). **l** Plasma iron levels 24h after CLP for mice treated with vehicle (2% DMSO) or compound UAMC-3203. The combined results of 2 independent experiments are shown (total n/condition from left to right is as follows: n = 5, 7, 9). The TNF experiments were performed on mice with a C57BL6/J background. Data were analyzed via unpaired two-tailed T testing (**a**-**h**, **k**, **l**,), and the Mantel-Cox test (**i**, **j**). Means and SEMs are represented. Source data are provided as a Source Data file.

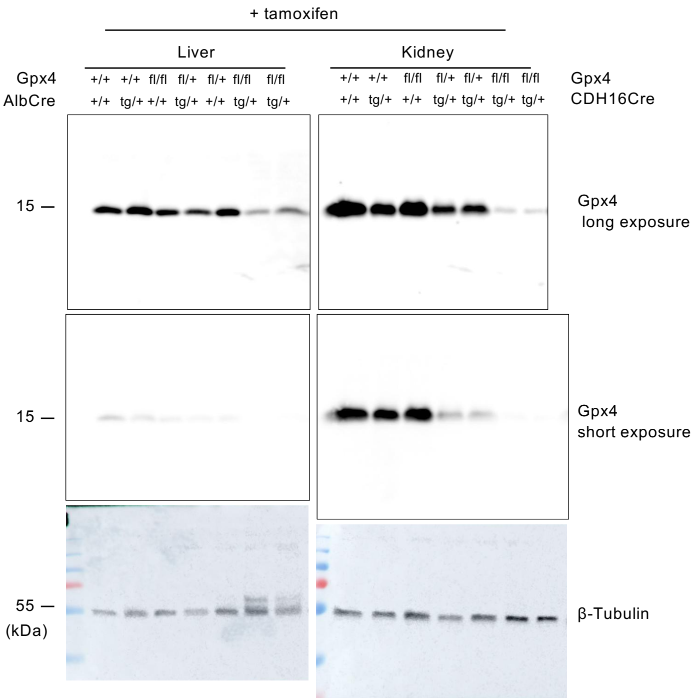

Figure 6a

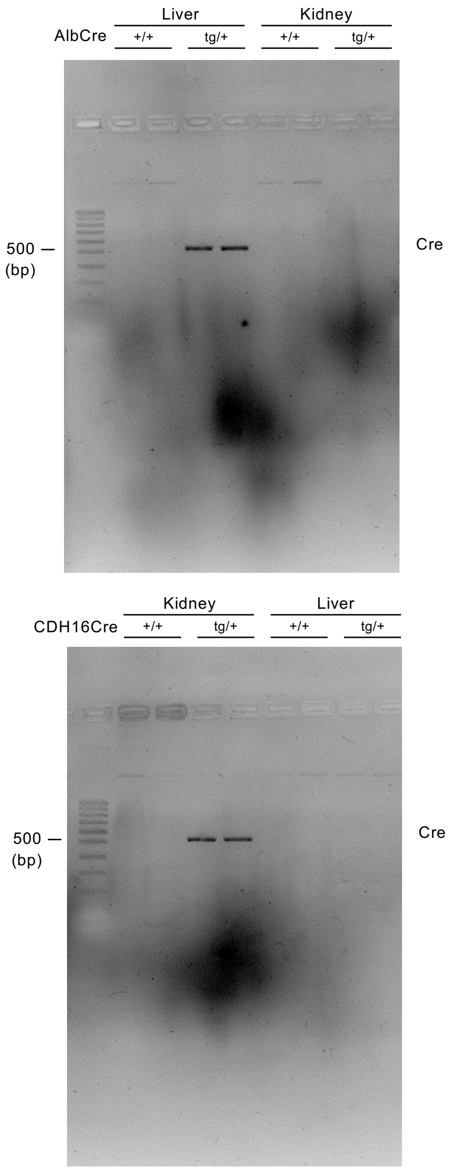

Figure 6b

Supplementary Fig. 10: Gels and blots source data. Uncropped and unprocessed version of the gels and blots presented in the supplementary figures.
