# Supplementary material for: Targeting ferroptosis protects against experimental (multi)organ dysfunction and death
Source: Nat Commun. 2022 Feb 24;13:1046. doi: 10.1038/s41467-022-28718-6 (PMC8873468; doi:10.1038/s41467-022-28718-6)
Supplement: Supplementary file 3 — Reporting Summary [file 41467_2022_28718_MOESM3_ESM.pdf]

nature portfolio

Corresponding author(s): Prof. Dr. Tom Vanden Berghe

Last updated by author(s): Nov 24, 2021

Reporting Summary

Nature Portfolio wishes to improve the reproducibility of the work that we publish. This form provides structure for consistency and transparency in reporting. For further information on Nature Portfolio policies, see our Editorial Policies and the Editorial Policy Checklist.

Statistics

For all statistical analyses, confirm that the following items are present in the figure legend, table legend, main text, or Methods section.

n/a	Confirmed	
☐	☒	The exact sample size (*n*) for each experimental group/condition, given as a discrete number and unit of measurement
☐	☒	A statement on whether measurements were taken from distinct samples or whether the same sample was measured repeatedly
☐	☒	The statistical test(s) used AND whether they are one- or two-sided<br>*Only common tests should be described solely by name; describe more complex techniques in the Methods section.*
☐	☒	A description of all covariates tested
☐	☒	A description of any assumptions or corrections, such as tests of normality and adjustment for multiple comparisons
☐	☒	A full description of the statistical parameters including central tendency (e.g. means) or other basic estimates (e.g. regression coefficient) AND variation (e.g. standard deviation) or associated estimates of uncertainty (e.g. confidence intervals)
☐	☒	For null hypothesis testing, the test statistic (e.g. *F*, *t*, *r*) with confidence intervals, effect sizes, degrees of freedom and *P* value noted<br>*Give P values as exact values whenever suitable.*
☒	☐	For Bayesian analysis, information on the choice of priors and Markov chain Monte Carlo settings
☒	☐	For hierarchical and complex designs, identification of the appropriate level for tests and full reporting of outcomes
☐	☒	Estimates of effect sizes (e.g. Cohen's *d*, Pearson's *r*), indicating how they were calculated

Our web collection on statistics for biologists contains articles on many of the points above.

Software and code

Policy information about availability of computer code

Data collection	No software was used for data collection
Data analysis	Sample size estimation was done by using G power 3.1.9.7. Depending on the analysis the following softwares were used: Zen v3.1, AxioVision v4.5 from Zeiss, QuPath-0.2.3, FlowJo v10.7, R version 3.6.1 (using the ggstatsplot package version 0.5.0, the package survival (v3.2.7) and survminer (v0.4.8)), Graphpad version 8.4.3 and Genstat version 20.

For manuscripts utilizing custom algorithms or software that are central to the research but not yet described in published literature, software must be made available to editors and reviewers. We strongly encourage code deposition in a community repository (e.g. GitHub). See the Nature Portfolio guidelines for submitting code & software for further information.

Data

Policy information about availability of data

All manuscripts must include a data availability statement. This statement should provide the following information, where applicable:

- Accession codes, unique identifiers, or web links for publicly available datasets
- A description of any restrictions on data availability
- For clinical datasets or third party data, please ensure that the statement adheres to our policy

The authors declare that the data supporting the findings of this study are available within the article and Supplementary Information Files. Source data are provided with this paper containing all unprocessed results and uncropped blots per figure.

Field-specific reporting

Please select the one below that is the best fit for your research. If you are not sure, read the appropriate sections before making your selection.

- [x] Life sciences
- [ ] Behavioural & social sciences
- [ ] Ecological, evolutionary & environmental sciences

For a reference copy of the document with all sections, see nature.com/documents/nr-reporting-summary-flat.pdf

Life sciences study design

All studies must disclose on these points even when the disclosure is negative.

Sample size	Sample size estimation was done by using G power 3.1.9.7.
Data exclusions	No data were excluded for statistical analysis
Replication	All data in the manuscript were independently and succesfully repeated at least once.
Randomization	For all in vivo experiments, mice were allocated randomly (within genotypical groups if applicable) to the different treatment groups. The clinical data required no allocation since there was no division in treatment groups.
Blinding	There was no group allocation of the patients, as this was a prospective cohort study without intervention of differing treatments. Investigators could thus not be blinded, as they provided the same standard of care to all patients. No bias could be introduced as all results were obtained by quantitative, objective measurements.

Reporting for specific materials, systems and methods

We require information from authors about some types of materials, experimental systems and methods used in many studies. Here, indicate whether each material, system or method listed is relevant to your study. If you are not sure if a list item applies to your research, read the appropriate section before selecting a response.

Materials & experimental systems

n/a	Involved in the study	
☐	☒	Antibodies
☒	☐	Eukaryotic cell lines
☒	☐	Palaeontology and archaeology
☐	☒	Animals and other organisms
☐	☒	Human research participants
☒	☐	Clinical data
☒	☐	Dual use research of concern

Methods

n/a	Involved in the study	
☒	☐	ChIP-seq
☐	☒	Flow cytometry
☒	☐	MRI-based neuroimaging

Antibodies

Antibodies used	anti-GPX4 (1:5000 Abcam; #ab125066), anti- ß-Tubulin coupled to HRP (1:10000, Abcam; #ab21058) and secondary anti-rabbit antibody (1:5000; VWR International, NA934)
Validation	Validation of these commercial antibodies can be found at the manufacturer's website. More specifically, Abcam provides an Abpromise guarantee for the use of ab125066 and ab21058 covering western blotting as application. For antibody VWR NA934 high sensitivity, precision and stability are demonstrated for western blotting on the antibody-specific data file availabe on the website.

Animals and other organisms

Policy information about studies involving animals; ARRIVE guidelines recommended for reporting animal research

Laboratory animals	Experiments were performed with C57BL/6N mice ordered from Janvier Labs, Ripk1ki mice, Ripk3-/-;Ppif-/-;Parp1-/-;Gpx4Tg/+ mice, Gpx4Tg/+ mice, Gpx4fl/cys R26CreERT2Tg/+ mice, Gpx4fl/fl AlbCreERT2Tg/+ mice and Gpx4fl/fl CDH16CreERT2Tg/+ mice. All mice used were between the age of 8 and 13 weeks, both males and females were used.
Wild animals	The study did not involve wild animals.
Field-collected samples	No field-collected samples were used.
Ethics oversight	All experiments were approved by the animal ethics committee of Ghent University, Antwerp University, or by ethic committees and local authorities in Dresden and conducted according to institutional, national and European animal regulations.

Note that full information on the approval of the study protocol must also be provided in the manuscript.

Human research participants

Policy information about studies involving human research participants

Population characteristics	The population consisted of 63,0% males age 51-70 with BMI 22-28 The reason for ICU admission was either medical (59.7%), elective surgery (7.2%) or urgent surgery (33.1%). On the first day of the study the mean SOFA score was 9 (7-11). For more detailed population characteristics the reader is referred to the original article: De Loor J, Decruyenaere J, Demeyere K, Nuytinck L, Hoste EA, Meyer E. Urinary chitinase 3-like protein 1 for early diagnosis of acute kidney injury: a prospective cohort study in adult critically ill patients. Crit Care. 2016 Feb 11;20:38. doi: 10.1186/s13054-016-1192-x. PMID: 26864834; PMCID: PMC4750195.
Recruitment	The prospective cohort study was conducted at the 22-bed surgical and 14-bed medical intensive care units (ICU) of Ghent University Hospital from September 2012 till August 2014. A potential bias typical (and unavoidable) to such ICU prospective studies is selection bias, i.e. not asking (legally authorized representatives of) the most critically ill patients for consent, by which these patients are not included.
Ethics oversight	The study was approved by the Ethical Committee of the Ghent University Hospital (Belgian registration number of the study: B670201213147), and conducted in accordance with the declaration of Helsinki and in compliance with the Good Clinical Practice Guidelines. All patients or their legally authorized representatives provided written informed consent.

Note that full information on the approval of the study protocol must also be provided in the manuscript.

Flow Cytometry

Plots

Confirm that:

- [x] The axis labels state the marker and fluorochrome used (e.g. CD4-FITC).
- [x] The axis scales are clearly visible. Include numbers along axes only for bottom left plot of group (a 'group' is an analysis of identical markers).
- [x] All plots are contour plots with outliers or pseudocolor plots.
- [x] A numerical value for number of cells or percentage (with statistics) is provided.

Methodology

Sample preparation	Dissociation of whole livers was performed as previously described. Briefly, after perfusion with PBS, livers were dissected, chopped finely, and subjected to GentleMACS dissociation followed by 20 min of incubation with 1 mg/mL Collagenase A (Roche; # 10103586001) and 10 U/mL DNase I (Roche; 10104159001) dissolved in RPMI (Gibco; #52400-025) at 37 °C, while shaking. Following a second round of GentleMACS dissociation, single cell suspensions were filtered over a 100 µm mesh filter and centrifuged (5 min, 400 g) with an excess of PBS. Any remaining red blood cells were lysed by resuspension in ACK buffer (Lonza; #10-548E) for 3 min, after which the cells were washed in PBS, further filtered over a 40 µm mesh filter and centrifuged once more at 400 g for 5 min. To make a single cell suspension of kidney, the tissue was perfused with PBS, both kidneys were dissected, chopped finely, and subjected to a 30 min enzymatic digestion with Collagenase type 1 (Sigma-Aldrich, #C-9891) dissolved in DMEM (Invitrogen; # 41965-039) at 37 °C. The remaining tissue was then gently disrupted by pipetting (25 mL pipet) and filtered over a 70 µm mesh filter. After the suspension was allowed to settle for 5 min, the upper part, not containing any fragments, was collected. The process of pipetting and filtering the suspension was repeated twice (10 mL and 5 mL pipet respectively) for the remaining settled cells in fresh DMEM. The pooled suspensions obtained were centrifugated (10 min, 1200 g) and the pellet was resuspended in ACK lysis buffer for 5 min before being centrifuged with an excess amount of DMEM once more at 1200 g for 10 min.
Instrument	Fluorescence intensity was measured using Fortessa LSRII in the B530 channel.
Software	Data analysis of flow cytometry experiment was performed using the FlowJo 10.7 software.
Cell population abundance	The FACS sorting was not performed. For flow cytometry experiments, minimum 10000 cells were analyzed after the initial gating (see below).
Gating strategy	Gating strategy: 1. FSC-A vs SSC-A: exclusion of the debris based on the size of the particles. 2. FSC-A vs FSC-H and SSC-A and SSC-H: exclusion of cell doublets based on the proportional increase of the area and height of the peak in both forward and site scatter. 3. Exclusion of dead cells by the permeability marker (DRAQ7).

- [x] Tick this box to confirm that a figure exemplifying the gating strategy is provided in the Supplementary Information.
